# ERCC6L2-Associated Inherited Bone Marrow Failure Syndrome: A Croatian Experience

**DOI:** 10.7759/cureus.76957

**Published:** 2025-01-05

**Authors:** Izabela Kranjcec, Nusa Matijasic Stjepovic, Katarina Vulin, Maja Pavlovic, Toni Matic, Roberta Sarkanji-Golub, Gordana Jakovljevic, Jelena Roganovic

**Affiliations:** 1 Department of Oncology and Hematology, Children's Hospital Zagreb, Zagreb, HRV; 2 Department of Medical Genetics and Reproductive Health, Children's Hospital Zagreb, Zagreb, HRV; 3 Department of Pediatrics, University Hospital Centre Zagreb, Zagreb, HRV; 4 Department of Medical and Laboratory Genetics, Endocrinology and Diabetology, Cytology Unit, Children's Hospital Zagreb, Zagreb, HRV

**Keywords:** bone marrow failure syndrome, genetic testing, hematopoetic myelodysplasia, leukopenia, thrombocytopenia

## Abstract

Inherited bone marrow failure syndromes (IBMFS) are often misdiagnosed or lately diagnosed despite thorough medical assessment. Genomic investigations have largely facilitated correct diagnosis and enabled effective management in children with IBMFS. We present two unrelated adolescent females with unexplained prolonged bicytopenia, unremarkable medical history and normal physical findings who were diagnosed with a rare non-classical *ERCC6L2*-associatedIBMFS. *ERCC6L2*-associated disease has been so far frequently related to neurodevelopmental delay and consanguinity and, most importantly, recognized as a predisposition syndrome to myeloid malignancies. Despite the same genetic findings, the patients experienced remarkably different clinical courses: over a decade of stable disease versus rapid progression to myelodysplasia requiring allogeneic stem cell transplant. We highlight the importance of early recognition and active surveillance in patients with bi-allelic *ERCC6L2 *variants.

## Introduction

Inherited bone marrow failure syndromes (IBMFS) are a heterogeneous group of rare disorders that mainly present as prolonged uni, bi, or tri-lineage cytopenia with/without macrocytosis, both in children and adults. The diagnosis is often challenging, as 40% of cases do not exhibit distinctive physical anomalies and a positive family history [1]. However, due to the high predisposition for myelodysplastic syndrome (MDS) and malignancy, predominately acute myeloid leukemia (AML), timely recognition and directed management are crucial [2]. Differential diagnoses, apart from the MDS itself, include infections, nutritional deficiencies, and metabolic disorders [1]. Genetic testing, including the next-generation sequencing (NGS), is a helpful complementary diagnostic tool, especially in atypical presentations. We hereby present two unrelated cases of excision repair cross-complementing six like two(*ERCC6L2*)-associated-IBMFS, with a similarly unremarkable medical history and lacking specific somatic features but showing a different clinical course.

## Case presentation

Case 1 

ERCC6L2-Associated Disease, an Example of a Prolonged, Stable Clinical Course

A 13-year-old girl was referred to the pediatric hematologist due to mild thrombocytopenia lasting for seven years. Thrombocytopenia was an accidental finding in a routine laboratory work-up performed by the primary care practitioner, as she did not have any complaints at the time nor during the entire observation period. Her personal and family history were unremarkable, as was the physical examination. Complete blood count (CBC) revealed a slightly reduced platelet count and increased mean corpuscular volume (MCV) (Table 1).

**Table 1:** Patients' characteristics. CBC: complete blood count.

Variables	Patient 1	Patient 2
Sex	Female	Female
Age	13 years	15 years
Medical history	Unremarkable	Unremarkable
Consanguinity	None	None
Physical appearance	Normal	Normal
CBC	At the time of diagnosis	At the time of diagnosis
Hemoglobin (g/L)	129	128
Mean corpuscular volume (fL)	103.4	98.2
Platelets (x10^9^/L)	96	68
Leukocytes (x10^9^/L)	5.35	3.0
Bone marrow	At the time of diagnosis	At the time of diagnosis
Cytology	Hypocellularity, macro erythroblasts changes, no maturation arrest	Hypocelullarity, no dysplastic changes, no maturation arrest
Histopathology	Moderate hypocellularity (10-50 % hematopoiesis left), dysplastic changes in the form of micro megakaryocytes	Severe hypocellularity (20% of hematopoiesis left), dysplastic changes in the form of micro megakaryocytes
Cytogenetics	46, XX	46, XX
Bone marrow	One-year follow-up	One-year follow-up
Cytology	Unchanged	Hypocellularity, myelodysplastic changes prior to transplant: dyserythropoietic, dysgranulocytopoiesis
Histopathology	Unchanged	Hypocellularity (35% of hematopoiesis left)
Cytogenetics	Unchanged	5q31 deletion
Genetic findings	*ERCC6L2(NM_020207.7): c.1930C>T, p.(Arg644Ter)* homozygosity	*ERCC6L2(NM_020207.7): c.1930C>T, p.(Arg644Ter)* homozygosity
Treatment	Close follow-up	Allogeneic stem cell transplant
Outcome	No symptoms; mild thrombocytopenia and leukopenia	Normal CBC

**Figure 1:**
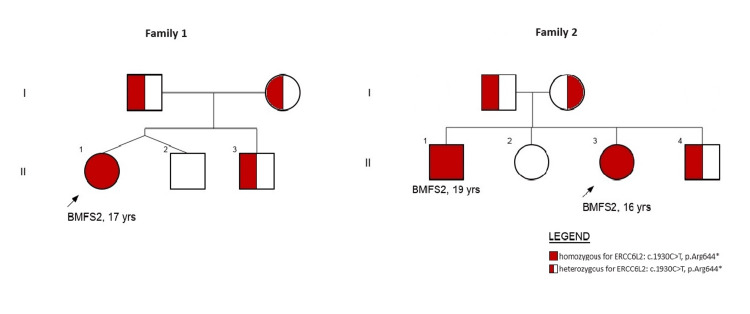
Patients' family genetic testing results

Meanwhile, seven months after the referral, leukocytopenia (leukocytes 3.5x10^9^/L) also emerged. Bone marrow examination showed reduced cellularity with no cytogenetic abnormalities (Table 1). Due to the persistent bicytopenia, after a four-year follow-up, genetic testing was performed, identifying a homozygous nonsense variant, ERCC6L2 c.1930>T, p.(Arg644*). At the age of 18 years, the girl remains under close clinical and laboratory monitoring. She continues to be transfusion-independent and shows no increased susceptibility to infections. Genetic counseling and testing were provided (Figure 1).

Case 2

*ERCC6L2-Associated Disease, an Example of Rapid Progression to Myelodysplasia Necessitating Allogeneic Stem Cell Transplant* 

A 15-year-old girl presented to the pediatric hematologist with mild leukopenia and moderate thrombocytopenia, accompanied by mild macrocytosis, three weeks following the SARS-CoV-2 infection (Table 1). She had no complaints. Apart from several hematomas on the lower extremities, her physical appearance was normal. The personal and family history was unremarkable. Bone marrow examination showed significantly reduced cellularity. Due to the persistent bicytopenia, seven months after the initial presentation, genomic evaluation was performed, showing a homozygous nonsense variant, *ERCC6L2* c.1930>T, p.(Arg644*). One year after the initial presentation, her CBC remained stable. In the subsequent bone marrow aspirate, 5q31 deletion was detected, guiding the patient's management toward an allogeneic stem cell transplant. Immediately prior to transplant, additional myelodysplastic features in the bone marrow were observed (Table 1). A matched unrelated donor (MUD) hematopoietic stem cell transplantation was performed. The conditioning regimen included fludarabine/busulfan/thiotepa protocol. Due to the increased risk of graft-versus-host disease (GVHD), GVHD prophylaxis consisted of pre-transplant anti-thymocyte globulin (ATG), post-transplant cyclophosphamide, followed by cyclosporine and mycophenolate mofetil. Engraftment occurred three weeks after HSCT. Acute skin GVHD responded to steroids. Genetic evaluation was performed prior to the transplant. The family genetic testing results are presented in Figure 1. Segregation analysis revealed one sibling with bi-allelic *ERCC6L2* variants without a previous diagnosis of hematological disorder.

## Discussion

*ERCC6L2* is a recently discovered gene linked to the development of hematological diseases, encoding a centromeric protein involved in chromatin unwinding as well as DNA repair, recombination, and translocation. Bi-allelic germline variants in *ERCC6L2* are associated with bone marrow failure syndrome two and increase the risk of developing myeloid malignancies in affected individuals. Homozygous pathogenic variants in *ERCC6L2* were initially identified in two consanguineous families, and the affected children had developmental delay and microcephaly in addition to bone marrow failure [3].

We describe the first two patients from Croatia with homozygous nonsense variant *ERCC6L2* c.1930C>T, p.Arg644*. The kinship between these two families and the consanguinity of the parents of our patients are not confirmed. Therefore, the variant could be more frequent in the Croatian population. This variant (also known as *ERCC6L2* (NM_020207.5): c.1963C>T, p.Arg655*) has been reported in the homozygous state in patients with IBMFS [3-6]. Tummala et al. identified the same variant in a French boy born to consanguineous parents. He exhibited bone marrow failure affecting three lineages and also presented with additional features [3]. Zhang et al. described a French patient with similar characteristics, including the age of onset, consanguineous parents, and disease features. This raised the possibility that the patient described by Zhang et al. could be the same as the one reported by Tummala et al. and later by Bluteau et al. [4,5]. Our patients, both homozygous for this variant, as well as the brother of one of them with the same genotype, did not exhibit microcephaly, developmental delay, or dysmorphic features. Other studies on *ERCC6L2* have also not reported extra-hematopoietic manifestations as part of the disease phenotype [5-9]. Therefore, these manifestations may be attributed to the additional pathogenic variants in the previously reported consanguineous families.

To date, more than 50 patients with bi-allelic *ERCC6L2* mutations and bone marrow failure have been reported in the literature [3-6, 8-10]. Most patients carry bi-allelic loss-of-function variants, including our two patients. However, a clear genotype-phenotype correlation has not been established in these cases. In the largest cohort of 52 patients with bi-allelic *ERCC6L2* variants reported by Hakkarainen et al., the most frequent initial manifestation was hypocellular bone marrow with cytopenia, observed in nearly two-thirds of patients, similar to our patients [6]. The median age at presentation for patients with bone marrow failure in the cohort was also comparable to the age at onset in our patients. The second most common hematological presentation in the aforementioned cohort was MDS/AML, observed in 29% of cases, while 10% of patients initially presented without symptoms. However, myelodysplasia was identified in one of our patients during the pre-transplant work-up, only two months after the occurrence of the oncogenetic mutation that directed her management toward an allogeneic transplant. According to Douglas et al., *ERCC6L2*-driven hematological malignancies harbor additional somatic tumor protein 53 (TP53) mutations already in the bone marrow failure phase [9]. Although TP53 mutations were not detected in our patients, further surveillance is mandatory.

The parents of the patients are typically carriers of the pathogenic variant, and genetic testing of apparently healthy siblings is essential since they are potential stem cell donors. Siblings with bi-allelic pathogenic variants must be included in hematological monitoring, which should include not only a CBC but also bone marrow analysis and screening for TP53 mutations [6]. This approach was implemented in the management of our second patient’s older brother, who was referred to the adult hematologist for close monitoring.

## Conclusions

The *ERCC6L2*-associated disease has been so far frequently related to neurodevelopmental delay and consanguinity and recognized as a predisposition syndrome to myeloid malignancies. We present two cases of unrelated adolescent females with persistent bicytopenia, unremarkable medical history and normal physical features, who were the first two patients from Croatia in whom the homozygous nonsense variant *ERCC6L2* c.1930C>T, p.Arg644* was found, most likely a frequent variant in the Croatian population. Despite the same genetic findings, the patients experienced remarkably different clinical course. In conclusion, we emphasize the importance of genetic testing in patients with cytopenias without apparent syndromic features, as this can facilitate early recognition and ensure appropriate active surveillance in patients with bi-allelic *ERCC6L2* variants.
